# Evidence of bromethalin toxicosis in feral San Francisco “Telegraph Hill” conures

**DOI:** 10.1371/journal.pone.0213248

**Published:** 2019-03-18

**Authors:** Fern Van Sant, Sayed M. Hassan, Drury Reavill, Rita McManamon, Elizabeth W. Howerth, Mauricio Seguel, Richard Bauer, Kathy M. Loftis, Christopher R. Gregory, Paula G. Ciembor, Branson W. Ritchie

**Affiliations:** 1 For the Birds, San Jose, California, United States of America; 2 Center for Applied Isotope Studies, University of Georgia, Athens, Georgia, United States of America; 3 Zoo/Exotic Pathology Service, Carmichael, California, United States of America; 4 Zoo and Exotic Animal Pathology Service, Infectious Diseases Laboratory and the Department of Pathology, University of Georgia, Athens, Georgia, United States of America; 5 Department of Pathology, College of Veterinary Medicine, University of Georgia, Athens, Georgia, United States of America; 6 Infectious Diseases Laboratory, University of Georgia, Athens, Georgia, United States of America; 7 New Materials Institute, University of Georgia, Athens, Georgia, United States of America; Charles University, CZECH REPUBLIC

## Abstract

During 2018, four free-ranging conures, from a naturalized flock in San Francisco, presented with a characteristic set of neurologic signs that had been reported in other individuals from this flock. The cause of morbidity or mortality in historic cases has not been identified. From these four subjects, fresh feces were collected during their initial days of hospitalization and submitted to the University of Georgia Infectious Diseases Laboratory and Center for Applied Isotope Studies for bromethalin and desmethyl-bromethalin quantitation. Using High Performance Liquid Chromatography, the laboratory detected bromethalin, a non-anticoagulant, single-dose rodenticide, in fecal samples from three subjects; half of these samples were also positive for desmethyl-bromethalin, bromethalin’s active metabolite. In three subjects that died, the UGA laboratory screened brain and liver samples and found bromethalin in all samples; desmethyl-bromethalin was detected in all but one brain sample, which was below the detection limit. Our findings suggest the conures are more resistant to bromethalin than are other species in which bromethalin has been studied, and/or that the conures may be ingesting the toxin at a sublethal dose. More data is needed to better assess the long-term effects of bromethalin on animals exposed at the subacute/chronic levels, and also to better understand the compartmentalization of bromethalin and desmethyl-bromethalin in a wider variety of species.

## Introduction

Since 1989 [1], a well-established flock of interbreeding feral conures has grown and extended its range both northward, along San Francisco, California, USA’s Embarcadero waterfront to the Presidio, and southward to Brisbane. The flock contains a mix of mitred (*Aratinga mitrata*), red-masked (also known as red-headed or cherry-head; *A*. *erythrogenys)* and red-fronted (also known as scarlet-fronted; *A*. *wagleri*) conures. The birds range in very populated areas; they commonly interact with local residents and tourists.

Since at least 1999 [2], there have been anecdotal and veterinary reports of a neurologic condition affecting many dozens of these conures. The condition appears to be unique to this flock, as we have found no reports of a similar condition from other naturalized conure flocks. The City of San Francisco prohibited feeding the birds when early instances of the condition were attributed to inappropriate diets. Most of the early cases were attributed to trauma, as several birds had broken bones and most had substantial bruising on their beaks and faces. Over time, a primary causative agent, leading to the traumatic injuries, was suspected due to a characteristic set of neurologic clinical signs that included ataxia, circling, seizures, and tumbling that most birds displayed to varying degrees. As the number and severity of cases increased, Mickaboo Companion Bird Rescue (https://mickaboo.org/), of San Jose, California, USA, committed the resources necessary to systematically evaluate the cause of death in affected conures. From 2013 through 2017, 15 subjects presented with these characteristic neurologic signs; all died and were submitted for complete necropsy and histological evaluation with the intent of identifying a causative agent. Postmortem evaluations of these historic subjects were handled by two pathology services. The Zoo/Exotic Pathology Service, in greater Sacramento, California, USA, conducted seven necropsies (n = one from 2013; six from 2017). The Infectious Diseases Laboratory, Zoo and Exotic Animal Pathology Service, at the University of Georgia, Athens, Georgia, USA, conducted eight necropsies (n = two from 2013; two from 2014; one from 2015; three from 2016).

Eight historic subjects (n = three from 2013; five from 2017) were found negative (antigen, antibody or nucleic acid) for common viruses and organisms that are associated with neurologic disease in parrots, including avian paramyxoviruses 1, 2 and 3; West Nile virus, avian bornavirus, *Chlamydia spp*., *Sarcocystis spp*. [3], *Baylisascaris spp*., and toxoplasmosis. In three subjects from 2017, brain, liver or serum samples were screened for lead and zinc, as well as other minerals and toxins; all were within normal limits for tested analytes. External laboratories did not detect bromethalin and/or desmethyl-bromethalin, which we had suspected as a potential etiology of the clinical signs since 2013, in five subjects tested postmortem (one per 2013, 2016, 2017; two from 2018). One consistent histologic finding in these historic subjects was a characteristic vacuolar degeneration of cerebellar and brain stem white matter, as well as other white-matter tracts, which has been described with bromethalin toxicosis in mammals [4–9]. All historic necropsies and diagnostic sample collections were performed on animals that had been treated with supportive care for weeks to months. Due to bromethalin’s reported 5.6 day half-life in plasma of other species [4], we theorized that any residual bromethalin or desmethyl-bromethalin in samples may have degraded or been below the detection limit by the time samples were collected. None of these historic subjects yielded enough sample (brain, liver, feces) for us to test again for this study; it is not possible to attribute the morbidity and mortality from these cases to a single cause.

Bromethalin is an odorless, potent neurotoxic rodenticide, developed by Lilly Research Laboratories in the 1970s [10–11] and registered with the EPA in 1984 [12]. It has been available to consumers since 1985 [13] and is designed to kill with a single ingestion. Bromethalin has been studied in only eight species (see Table 1) and its lethality has been well described [4–9]. Only the guinea pig (*Cavia porcellus*) is known to be functionally resistant, due to its inability to metabolize bromethalin into the more lethal desmethyl-bromethalin. The effects of sublethal doses in animals are described as: “No convulsions are seen but rather animals become lethargic, display a hind leg weakness due to loss of muscle tone, and paralysis. A decreased response to tactile stimuli can also be observed” [4]. These clinical changes associated with sublethal doses are similar to the neurologic signs displayed by our 15 historic subjects, as well as by four subjects that presented in 2018. Due to the consistency of lesions found in our historic subjects, and our inability to detect infectious diseases or other toxins, we undertook this study to determine if bromethalin toxicosis was a factor in the morbidity and mortality of four affected conures that presented between June and September 2018.

**Table 1:** Median Oral Lethal Dose/Lethal concentration (LD_50_/LC_50_) of technical grade bromethalin in mammals and birds [4].

Rat (*Fischer 344*)	2.0mcg/g
Mouse (*Institute of Cancer Research*)	5.3mcg/g
Dog (*Canis familiaris*)	4.7mcg/g
Cat (*Felis catus*)	1.8mcg/g
Rhesus Monkey (*Macaca mulatta*)	5.0mcg/g
Rabbit (*Leporidae*)	13.0mcg/g
Adult Quail (*Coturnix coturnix*)	4.6mcg/g
Guinea pig (*Cavia porcellus*)	>1000mcg/g

## Materials and methods

### Ethics and funding statements

Mickaboo Companion Bird Rescue, a 501(c)3 non-profit organization, founded in 1996, paid for all of the diagnostic evaluations. No grant funding was used to support diagnostic work for this publication. No research animals were used in this study; diagnostic studies are not reviewed by the UGA Institutional Animal Care and Use Committee. The authors have no conflicts of interest to report. All authors on this study made contributions that led to our discovery.

The three subjects from 2018 that died were each necropsied and histologically examined by pathologists at the Zoo/Exotic Pathology Service, in greater Sacramento, California, USA.

Screening for bromethalin and desmethyl-bromethalin in the 2018 cohort was conducted by the UGA Laboratory for Environmental Analysis, part of the Center for Applied Isotope Studies. For sample controls, we collected and tested feces from captive psittacine birds that are kept in a controlled environment, at the University of Georgia College of Veterinary Medicine, and have no known exposure to bromethalin. Control samples of brain and liver, from chickens (*Gallus gallus*) with no known exposure to bromethalin or desmethyl-bromethalin, were salvaged during a scheduled necropsy of clinically normal control chickens from a UGA project not affiliated with this study. We report the findings of week two fecal samples from our subjects, because 1) this was the consistent clinical sample we had for comparison between these subjects, and 2) preliminary testing on historic cases had suggested that bromethalin could still be detected following two weeks of hospitalization.

We chose to test feces because free-ranging birds typically have very little adipose tissue for antemortem biopsy, and we can only take/test brain and liver samples from deceased subjects. Studies using radio-labeled bromethalin show the toxin is excreted primarily through the feces [13]. Fecal samples from the 2018 subjects in this study were collected by the veterinarians and staff at For the Birds, San Jose, California, USA, where the four subjects that presented with characteristic neurologic signs were each hospitalized in incubators on flannel bedding. Fecal samples were collected throughout each day as individuals produced feces; this practice continued daily until an individual died. In order to collect enough sample from each individual for testing, each subject’s sample was pooled weekly. All samples were placed in 30mL fecal specimen collection containers (manufactured by Grenier Bio-On, Kremsmünster, Austria) and labeled with the subject’s name and the subject’s week of hospitalization during which the feces was collected (e.g., “week two”). All samples were stored in a dark freezer at -14°C. Under these conditions, one gram of feces was frozen solid in 40 minutes. Smaller samples added to an already frozen sample collection tube froze in less than 10 minutes. Frozen samples were shipped overnight to the UGA Infectious Diseases Laboratory, where they were stored in a dark freezer at -20 to -30°C.

### Necropsy and histopathology

Complete post mortem examinations were performed by the Zoo/Exotic Pathology Service on three subjects from the 2018 cohort (see Table 2). Representative tissues sections from all organs were collected and fixed in 10% neutral buffered formalin. Paraffin-embedded tissues were sectioned at approximately 5 μm, mounted on glass slides, and stained with hematoxylin and eosin (HE). Sections of cerebrum, liver, kidney, intestines, bone, and skeletal muscle were collected with minimal exposure to light and stored in Corning 2 ml cryogenic vials in a dark freezer at -18°C. No immunohistochemical (IHC) testing for *Toxoplasma gondii*, *Sarcocystis spp*, or West Nile virus was performed on cerebrum, cerebellum and heart sections from the 2018 subjects necropsied by this service, based on negative results from these tests performed on historic cases (that presented from 2013 through 2017).

**Table 2:** Test results for study subjects, all of which displayed severe neurologic deficits upon initial presentation. Time under treatment is counted from the first day of hospitalization. Salient lesions are noted for necropsied subjects. Concentration of bromethalin or desmethyl-bromethalin detected is noted for positive test results; all screening was conducted by the UGA Center for Applied Isotope Studies Laboratory for Environmental Analysis.

Subject (Year)	Treatment time	Salient histologic lesions of the CNS	Testing (Sample)	
			Bromethalin	Desmethyl-bromethalin
1 (2018)	2 weeks		18.37 μg/g (Fecal)	Negative (Fecal)
**2** (2018)	2 weeks		4.06 μg/g (Fecal)	Negative (Fecal)
**2** (2018)	2 months	Vacuolar degeneration of cerebellar white matter.	11.91 μg/g (Brain) 9.68 μg/g (Liver)	0.258 μg/g (Brain) 0.283 μg/g (Liver)
**3** (2018)	2 weeks		0.8 μg/g (Fecal)	1.68 μg/g (Fecal)
**3** (2018)	3 months	Vacuolar degeneration of cerebellar white matter that extends into the brain stem.	0.283 μg/g (Brain) 10.84 μg/g (Liver)	*Below Detection Limit* (Brain) 6.19 μg/g (Liver)
**4** (2018)	2 weeks		*Below Limit of Quantitation* (Fecal)	0.28 μg/g (Fecal)
**4** (2018)	2 months	Vacuolar degeneration at the base of the folia and extending into the white matter of the folia.	0.03 μg/g (Brain) 0.21 μg/g (Liver)	0.05 μg/g (Brain) 0.03 μg/g (Liver)

### Liquid chromotography

#### Chemicals

Cyclohexane (EMD Millipore Corporation, Billerica, Massachusetts, USA) and methanol (VWR Chemicals, Radnor, Pennsylvania, USA) were HPLC grade solvents. Water was double deionized. Bromethalin standard was obtained as 100 mg/L ampoule in cyclohexane from Crescent Chemical Co., Inc., Islandia, New York, USA. Desmethyl-bromethalin standard, 98% purity, was obtained from Toronto Research Chemicals, Toronto, Ontario, Canada.

#### Apparatus

The HPLC used consisted of an SIL-10 AD Auto-injector, LC-10AD dual pump solvent delivery module, SCL-10A system controller, a DGU-14A degasser, and an SPD-10 AV UV-Vis detector (all are products of Shimadzu Corporation, Kyoto, Japan). Data collection and processing was done using Class-8000 LC/MS software, also from Shimadzu Corporation. Separation was done using Zorbax SB-C18 Rapid Resolution HT column 4.6 cm X 50 mm, 1.8 micron. The injected volume was 60 μl, UV detector set at 261 nm and the eluent was methanol-water (3:1) at a combined flow rate of 0.6 ml/minute. The retention times of desmethyl-bromethalin and bromethalin were 9.1 minutes and 21.4 minutes, respectively.

#### Extraction of biological samples

From each submitted specimen (liver, brain or fecal matter), about 0.5 g of sample was accurately weighed, transferred into 50 ml screw-capped polypropylene centrifuge tubes and placed in a vacuum desiccator to remove moisture. Samples were treated with 10 ml of cyclohexane and placed in an ultrasonic bath for 30 minutes, then centrifuged at 1500 rpm for 20 minutes. The clear supernatant layer was transferred to glass evaporation tubes while the residue was re-extracted and centrifuged for another two times using 5 ml of cyclohexane. The combined cyclohexane extracts were cautiously evaporated almost to dryness under a stream of nitrogen in a TurboVap LV concentration workstation. The residue was dissolved in 1 ml of methanol and analyzed by HPLC-UV for both bromethalin and desmethyl-bromethalin content.

#### Validation and quantitation

Method validation was based on stoichiometry of response to concentration for the standards and on running control extracts. In this respect, the mean area from replicate analysis for 1 μg/ml desmethyl-bromethalin is 10,377.0 with a standard deviation of 281.9. The mean area from replicated analysis for 1 μg/ml bromethalin is 2,443.7 with a standard deviation of 97.2. For both compounds, the coefficient of variation is less than 5%. Additionally, control extracts from brain and liver samples, from chickens with no exposure to bromethalin or desmethyl-bromethalin, and fecal samples from conures with no exposure to bromethalin or desmethly-bromthelain showed no response on the chromatogram at the retention time for either compound. The brain and liver samples were salvaged during a scheduled necropsy of clinically normal control chickens from a UGA project not affiliated with this study.

Quantitation was carried out on desmethyl-bromethalin and bromethalin standards in methanol. The lower limit of detection (LOD) was calculated by determining the signal-to-noise (s/n) ratio for standards of 0.5, 1.0, and 1.5 ppm of both compounds. These ratios were then interpolated to an s/n ratio of 3. The LOD for desmethyl-bromethalin was 3.48 ppb and the LOD for bromethalin was 7.68 ppb. The corresponding limits of quantitation (LOQ) were calculated by the formula LOQ = LOD x 3.3 [14]. The LOQ for desmethyl-bromethalin was 11.47 ppb and the LOQ for bromethalin was 25.35 ppb. Linear regression of standards provided *R*^2^ values of 0.997 for desmethly-bromethalin and 0.990 for bromethalin, demonstrating linearity for both compounds.

## Results

**Fig 1:**
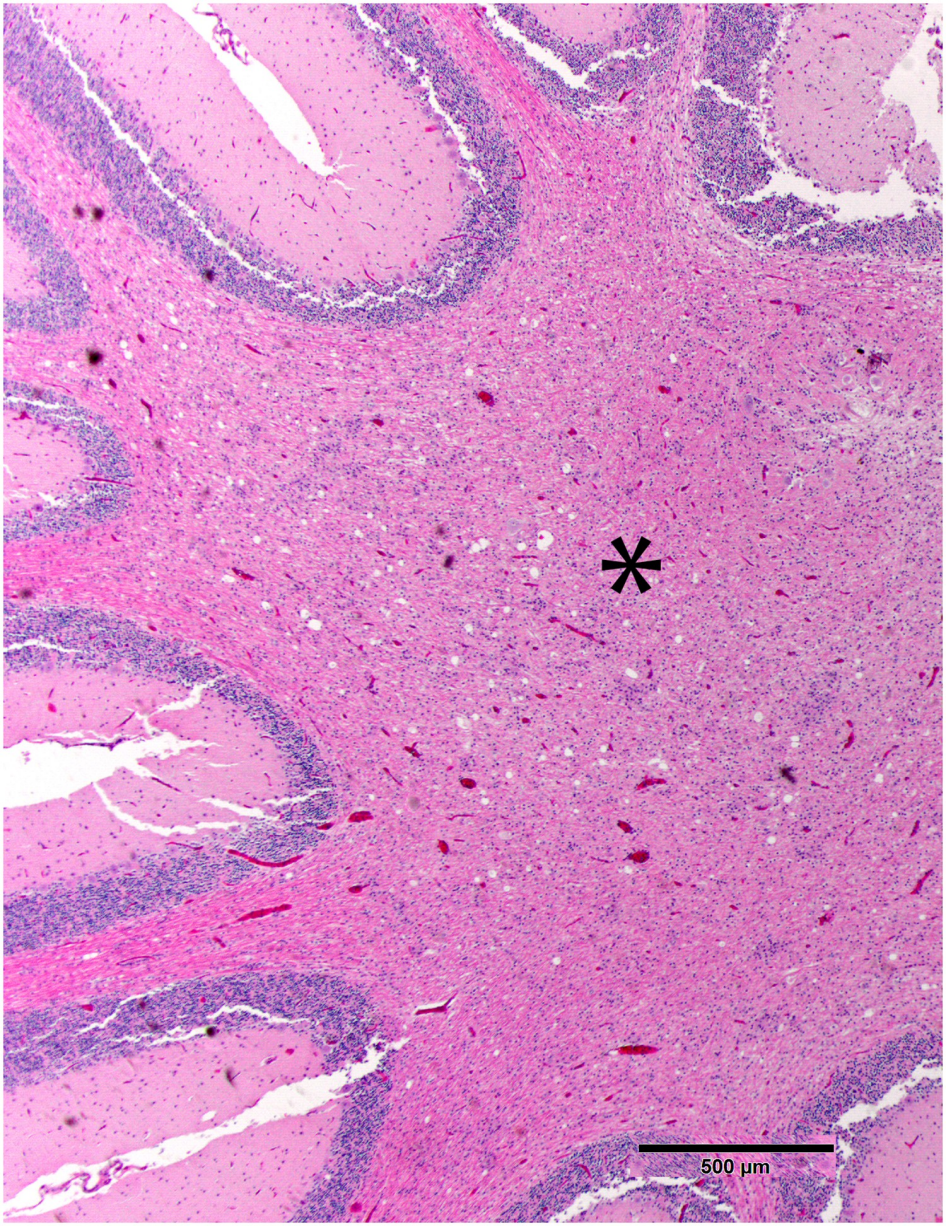
Prominent and consistent lesion, low magnification. The prominent and consistent lesion is of cerebellar white matter vacuolization (*) with increased cellularity due to gliosis. Fig 1 is from subject 2. HE stain. Bar = 500μm.

**Fig 2:**
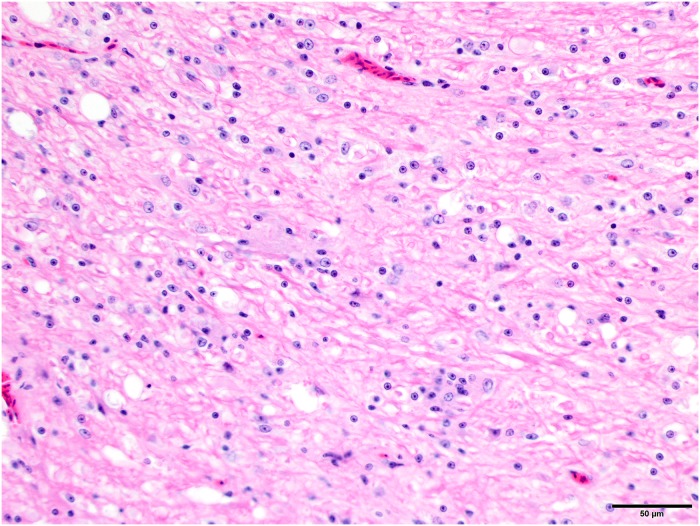
Prominent and consistent lesion, higher magnification. Higher magnification of the vacuolization in the white matter of the cerebellum and increased cellularity due to gliosis. Fig 2 is from subject 2. HE stain. Bar = 50 μm.

**Fig 3:**
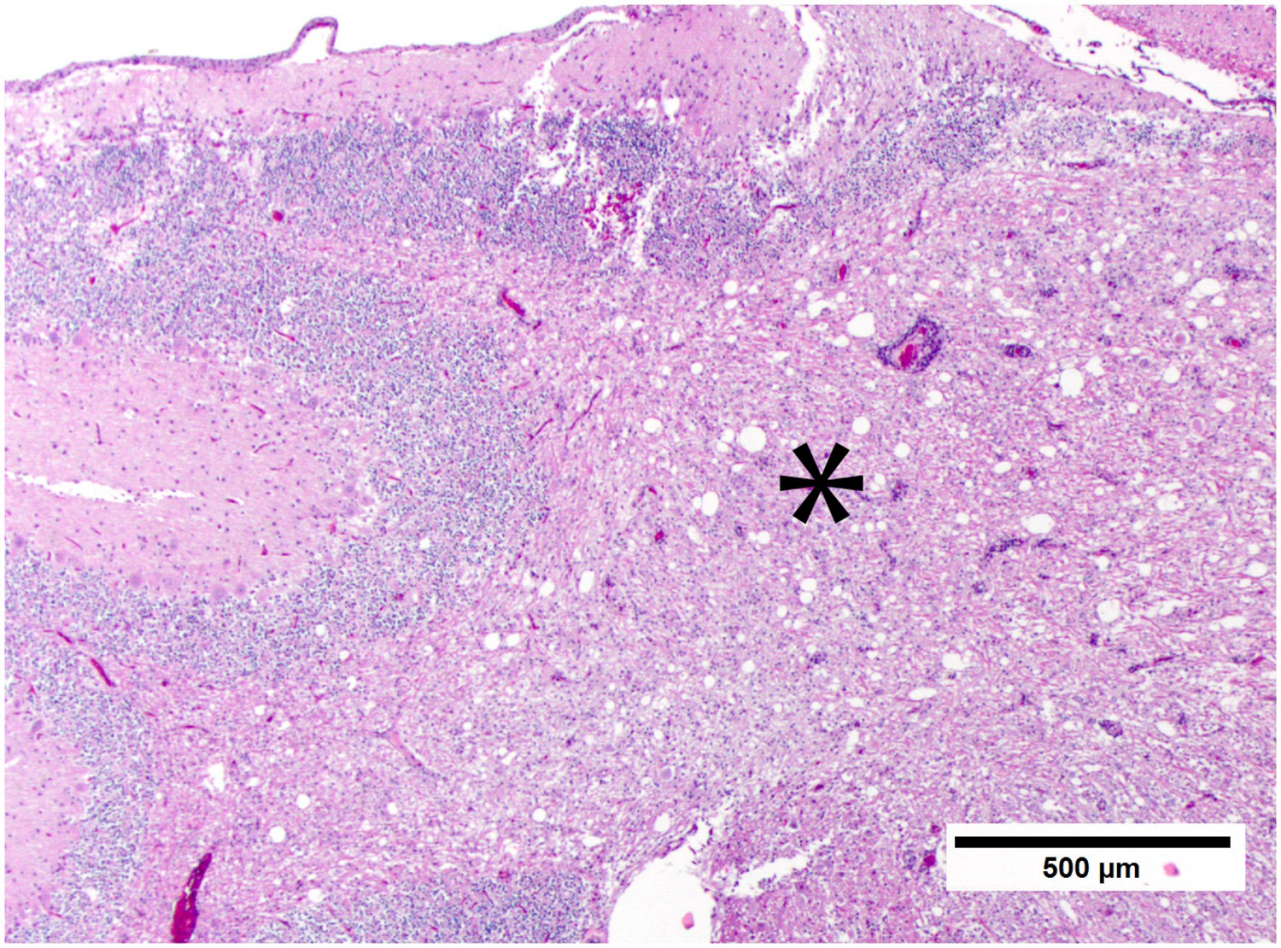
Prominent and consistent lesion, low magnification. The prominent and consistent lesion is of cerebellar white matter vacuolization (*) with increased cellularity due to gliosis. Fig 3 is from subject 3. HE stain. Bar = 500μm.

**Fig 4:**
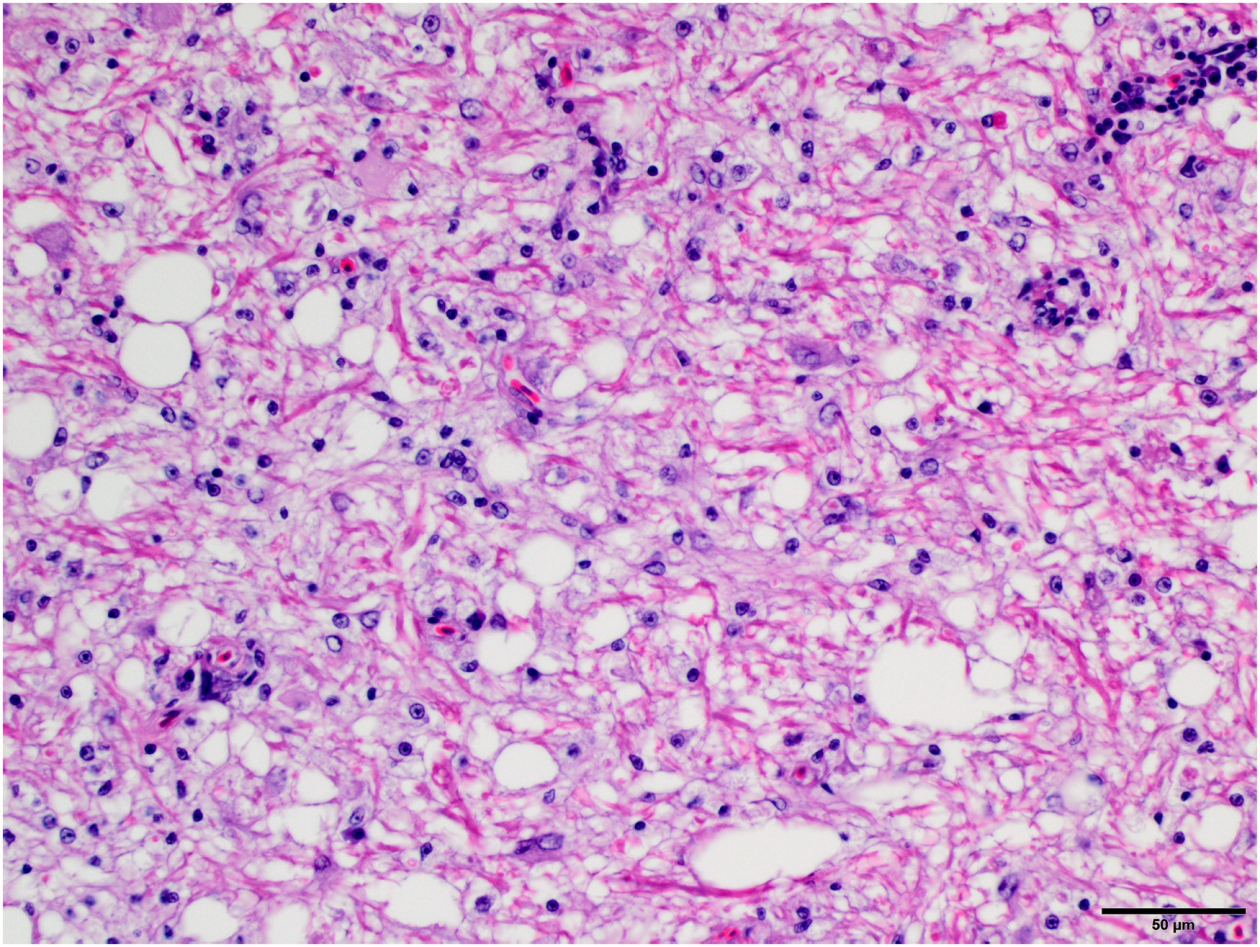
Prominent and consistent lesion, higher magnification. Higher magnification of the vacuolization in the white matter of the cerebellum and increased cellularity due to gliosis. Fig 4 is from subject 3. HE stain. Bar = 50 μm.

In 2018, San Francisco Department of Animal Care & Control transferred four recently rescued conures to For the Birds, in San Jose, California, USA, for veterinary care. To increase the likelihood of detecting bromethalin ingestion in these acute and severely affected subjects, feces was collected from each and submitted to the UGA Center for Applied Isotope Studies (CAIS), Athens, Georgia, USA, for bromethalin and desmethyl-bromethalin screening. Due to the progression of their neurologic deficits, subjects two and three were euthanized at two and three months, respectively, following initial hospitalization. Subject four died two months following initial hospitalization, after neurological signs progressed to the point that the subject could no longer self-feed. Salient histologic lesions of the CNS from the deceased subjects were consistent with those described in literature (see Figs 1–4). We detected bromethalin in samples from all four subjects from 2018. See Table 2 for all results.

## Discussion

The findings from our 2018 cohort suggest that conures may not metabolize bromethalin into desmethyl-bromethalin as effectively as this conversion occurs in the species studied and described in our literature review [4–5, 8–9, 15]. Because there is no pharmacokinetic data associated with bromethalin in conures, we were unable to confirm why our method detected desmethyl-bromethalin in half of the week two fecal samples from our 2018 subjects, but not in fecal samples from the other two subjects. Nor could we confirm why, weeks later, bromethalin was detected in the brains and livers from all deceased conures, and desmethyl-bromethalin was detected in all liver samples and all but one brain sample. However, variations in the concentration levels of these substances detected in our subjects would be expected to be dependent upon the time between ingestion by an individual and that individual’s presentation to the clinic. The LD_50_ of bromethalin varies by species with domestic felines being most susceptible and rabbits (*Leporidae*) being more resistant than canines or quail (*Coturnix coturnix*). Our data suggests that conures are more resistant to bromethalin toxicosis than rabbits, canines or quail. Whether this resistance is acquired or innate is not known, but should be better defined to understand potential ways to protect and/or treat collaterally affected species.

Our findings indicate it is likely that bromethalin contributed to the morbidity and mortality of these specific individuals from 2018, although the source of bromethalin remains unclear. As of early February 2019, subject 1 remained alive and was able to self-feed, but its ataxia had progressed. Whether the conures are directly ingesting bromethalin in a bait or, potentially, through accumulated toxin in soil or water remains to be determined. If other birds, or untested mammals, have similar resistance, then in theory they could accumulate the toxin with potential subsequent poisoning of predatory or scavenging species; relay toxicosis, or secondary poisoning, from bromethalin has not been proven. Efforts are now underway to map the locations where feral conures have been found by concerned citizens and animal control personnel to see if the source of the poison can be identified.

There is no specific antidote for bromethalin toxicosis or in-clinic diagnostic assay. Clinical signs and death have been documented in domestic canines and felines at much lower doses than the specified LD_50_ for each species [4]. Unfortunately, there are few therapies available for animals that ingest bromethalin. A lipid emulsion therapy proposed for accidental ingestion in pets may be possible in poisoned San Francisco conures, but early treatment is essential, and IV or IO administration might be challenging or nearly impossible in feral parrots. Diagnosis of bromethalin poisoning has proven to be challenging in mammals and even more so in avian species. When traces of the toxin are found, it is typically in adipose tissue. As most free-ranging avian species have limited stored fat, documentation of bromethalin toxicosis in free-ranging birds has been lacking. Although characteristic of bromethalin toxicosis, cerebellar and white-matter lesions can, and often do, result from nonspecific postmortem change.

Over the last 20 years, first-generation anticoagulants (Warfarin, diphacinone) were replaced with more potent second-generation anticoagulant rodenticides (SGARs) as rodents became increasingly resistant to the former. Reports of animal and human exposure to bromethalin increased following 2008 EPA mandates designed to decrease the use of SGARs like brodifacoum and bromadiolone, which are known to cause unintended secondary toxicosis in wildlife [12, 16–17]. The Pet Poison Helpline saw a 65% increase in bromethalin toxicosis cases reported between 2011 and 2014 [17]. The American Society for the Prevention of Cruelty to Animals said it received 2791 calls regarding exposure to bromethalin-based poisonings in 2015 [18]. There is supportive evidence that accumulation of sublethal amounts of SGARs in wildlife has contributed to mortality and chronic illness in both birds and mammals [12, 19–25]. The true extent of ancillary poisoning in unintended species by these replacement rodenticides may be impossible to ascertain due to lack of obvious chemical signatures and the reported short half-life of bromethalin in plasma. Our findings suggest that bromethalin and desmethyl-bromethalin quantification in fecal samples, using the modality employed by the UGA CAIS Laboratory for Environmental Analysis, may provide a valid screening assay for subjects with early clinical disease that process the toxin similarly to the conures, and therefore be a method for early detection of ingested bromethalin.

Our review of the available literature revealed no data on the clearance time of bromethalin or desmethyl-bromethalin in sublethally affected patients, or what conditions, other than light, may contribute to the decomposition of bromethalin [26]. There are two 13-week studies on the effect of subchronic or prechronic exposure to bromethalin in Sprague Dawley rats and beagles (*Canis familiaris*), but these regulatory documents were not accessible, so we do not know if these studies defined a half-life for bromethalin or desmethyl-bromethalin in tissues. [4, 12, 27]. In addition to the lack of information on the half-life for these substances in tissues, we faced multiple unknown variables including: what was ingested by our subjects, how much, when, and in what environment. The half-life of bromethalin in soil is 178 days where it is metabolized to desnitrobromethalin [12, 27–28], a substance about which the authors found nothing published. A 2009 review of bromethalin by the Thurston County, Washington, USA, Health Department noted that bromethalin is “high in hazard for persistence and bioaccumulation” and that it lacks a long-term risk assessment “…which creates a significant data gap…” [28].

To the scientific community at-large, we recommend that more data is needed to better assess the long-term effects of bromethalin on animals exposed at the subacute/chronic levels, and also to better understand the compartmentalization of bromethalin and desmethyl-bromethalin in a wider variety of species, including how both substances accumulate in tissues. We also believe more reliable methods for detecting bromethalin, as well as desmethyl-bromethalin, are needed, particularly methods for rapid detection of this toxin at subacute/chronic levels in species that may not process the organism as typically described. Such studies would help determine the risk factor for relay toxicosis from bromethalin and yield important data about the chemical’s potential long-term impact on the environment and wildlife.

## Supporting information

S1 Fig Linearity of desmethyl-bromethalin concentration. Calibration curve for desmethyl-bromethalin standards at 0.5, 1.0, and 1.5 ppm fitted with a linear model (*R*^2^ = 0.997).
(PNG)

S2 Fig Linearity of bromethalin concentration. Calibration curve for bromethalin standards at 0.5, 1.0, and 1.5 ppm fitted with a linear model (*R*^2^ = 0.990).
(PNG)

S3 Fig Chromatogram of methanol blank injection. Liquid chromatogram of a methanol blank injection using the same system settings used in the testing for bromethalin and desmethyl-bromethalin.
(TIF)

S4 Fig Chromatogram of detection of desmethyl-bromethalin standard. Liquid chromatogram of a 1 ppm desmethyl-bromethalin standard showing detection at 9.135 minutes.
(TIF)

S5 Fig Chromatogram of detection of bromethalin standard. Liquid chromatogram of a 1 ppm bromethalin standard showing detection at 21.371 minutes.
(TIF)

S6 Fig Chromatogram of control fecal sample. Liquid chromatogram of a control fecal sample showing no detection at either the desmethyl-bromethalin or bromethalin retention times.
(TIF)

S7 Fig Chromatogram of fecal sample. Liquid chromatogram of fecal sample from subject 1 showing detection of bromethalin at 21.261 minutes.
(TIF)

S8 Fig Chromatogram of control chicken brain sample. Liquid chromatogram of a chicken brain sample showing no detection at either the desmethyl-bromethalin or bromethalin retention times.
(TIF)

S9 Fig Chromatogram of conure brain sample. Liquid chromatogram of a brain sample from subject 4 showing detection of bromethalin at 21.9 minutes.
(TIF)

S10 Fig Chromatogram of control chicken liver sample. Liquid chromatogram of a chicken liver sample showing no detection at either the desmethyl-bromethalin or bromethalin retention times.
(TIF)

S11 Fig Chromatogram of a conure liver sample. Liquid chromatogram of a liver sample from subject 4 showing detection of bromethalin at 21.17 minutes.
(TIF)

S1 Video This subject was among 5 conures that presented for care in June and July 2017. It remained hospitalized until late fall 2017, when it was euthanized due to declining health. The ataxia and recumbency exhibited by this bird is typical of the late-stage neurological symptoms clinically documented by the authors on other conures examined during this study.
(MP4)

S2 Video This subject was also among 5 conures that presented for care in June and July 2017. It also remained hospitalized until late fall 2017, when it was euthanized due to declining health. The ataxia and recumbency exhibited by this bird is typical of the late-stage neurological symptoms clinically documented by the authors on other conures examined during this study.
(M4V)
