# Supplementary figures and images for: Evidence of bromethalin toxicosis in feral San Francisco “Telegraph Hill” conures
Source: PLoS One. 2019 Mar 18;14(3):e0213248. doi: 10.1371/journal.pone.0213248 (PMC6422264; doi:10.1371/journal.pone.0213248)

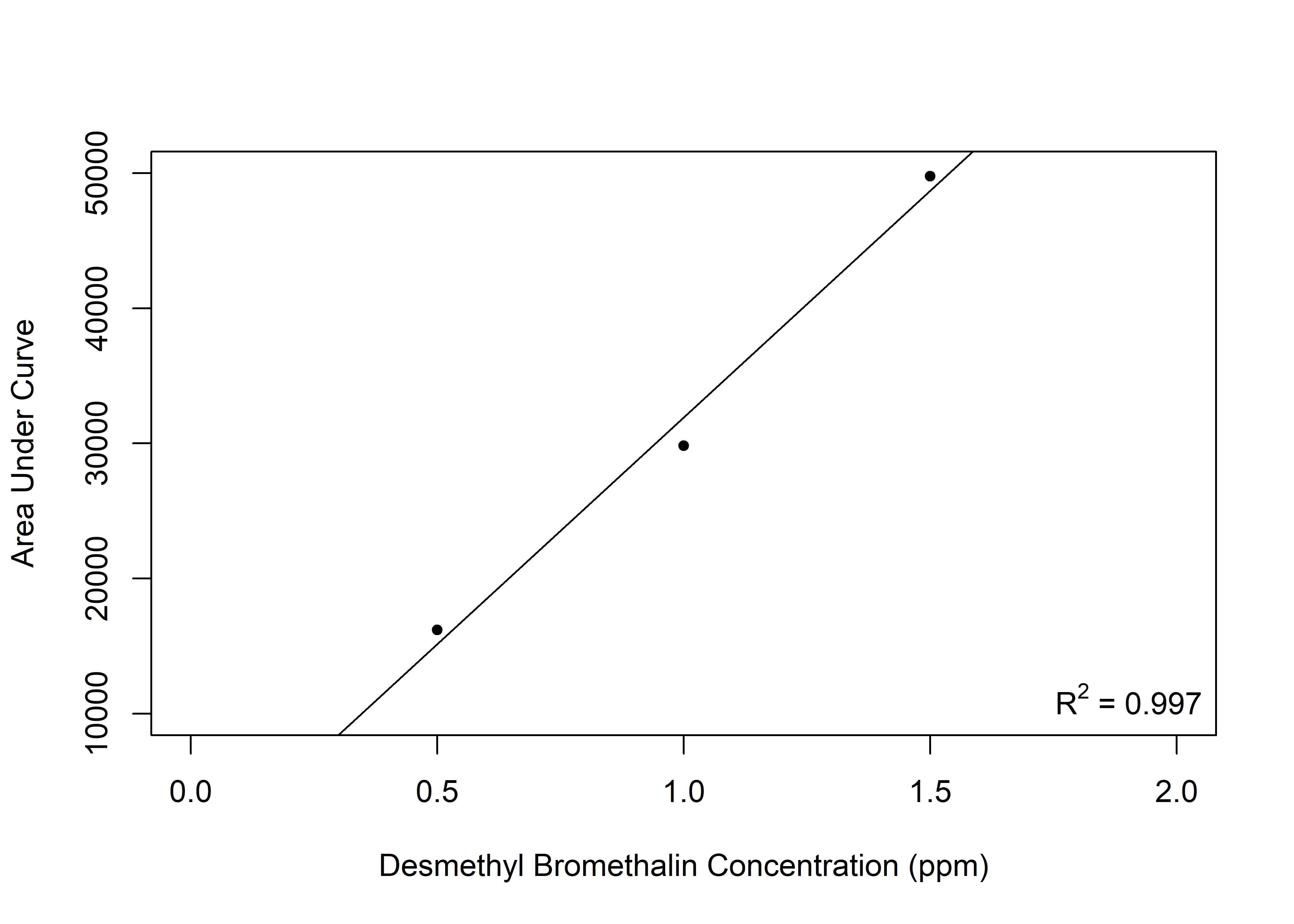

Supplement: S1 Fig — Calibration curve for desmethyl-bromethalin standards at 0.5, 1.0, and 1.5 ppm fitted with a linear model (R2 = 0.997). (PNG) [file pone.0213248.s001.png]

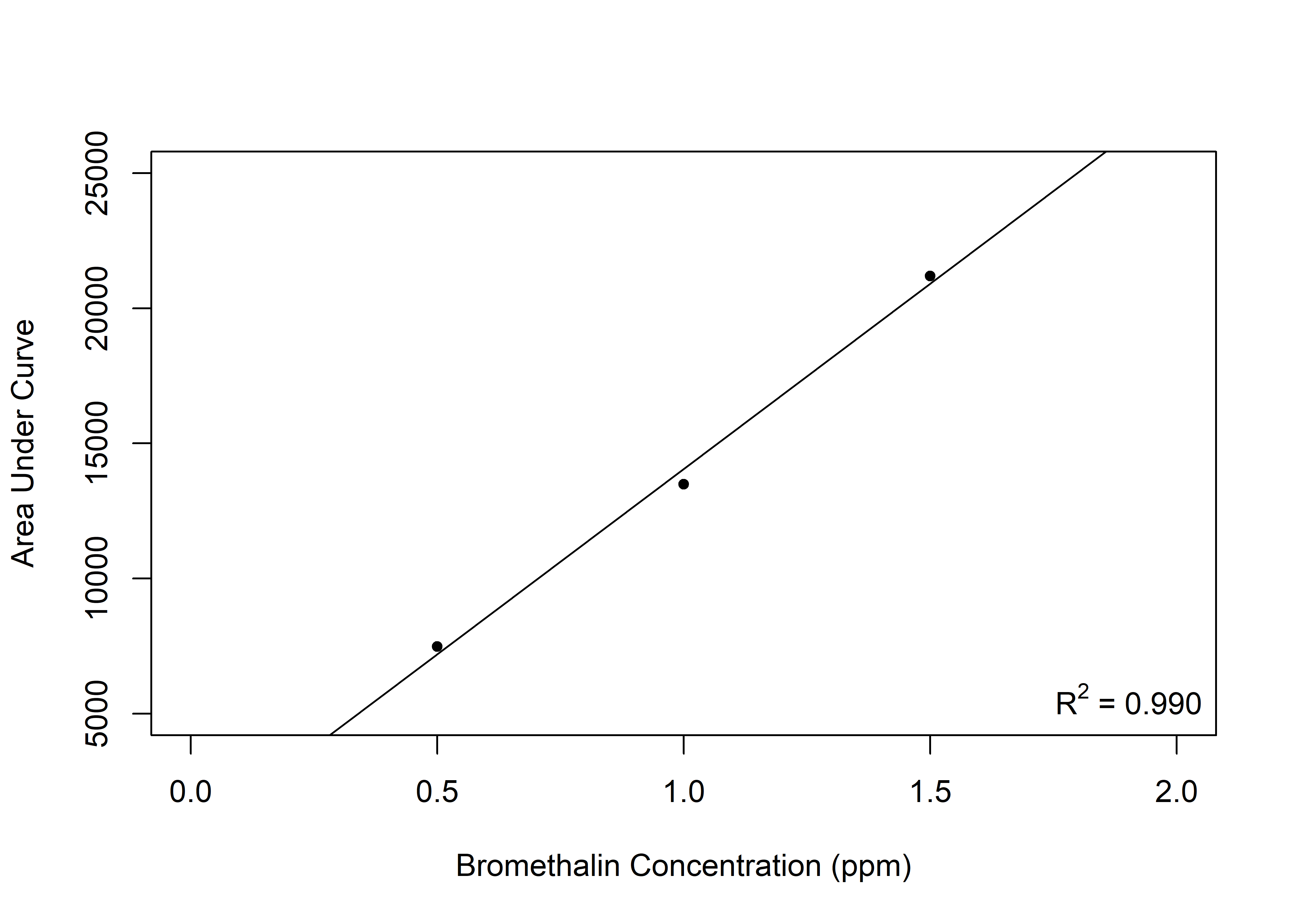

Supplement: S2 Fig — Calibration curve for bromethalin standards at 0.5, 1.0, and 1.5 ppm fitted with a linear model (R2 = 0.990). (PNG) [file pone.0213248.s002.png]

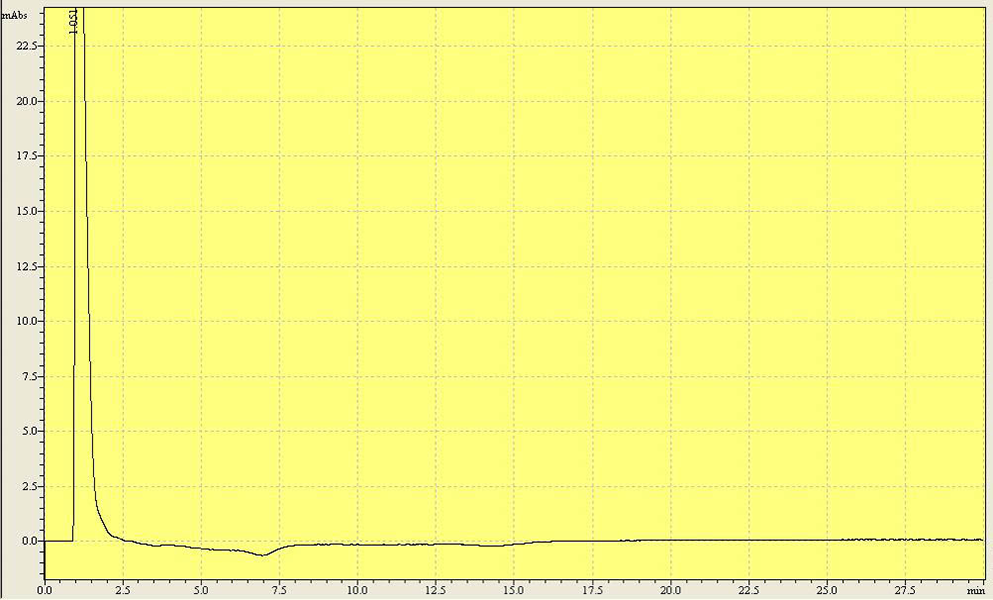

Supplement: S3 Fig — Liquid chromatogram of a methanol blank injection using the same system settings used in the testing for bromethalin and desmethyl-bromethalin. (TIF) [file pone.0213248.s003.tif]

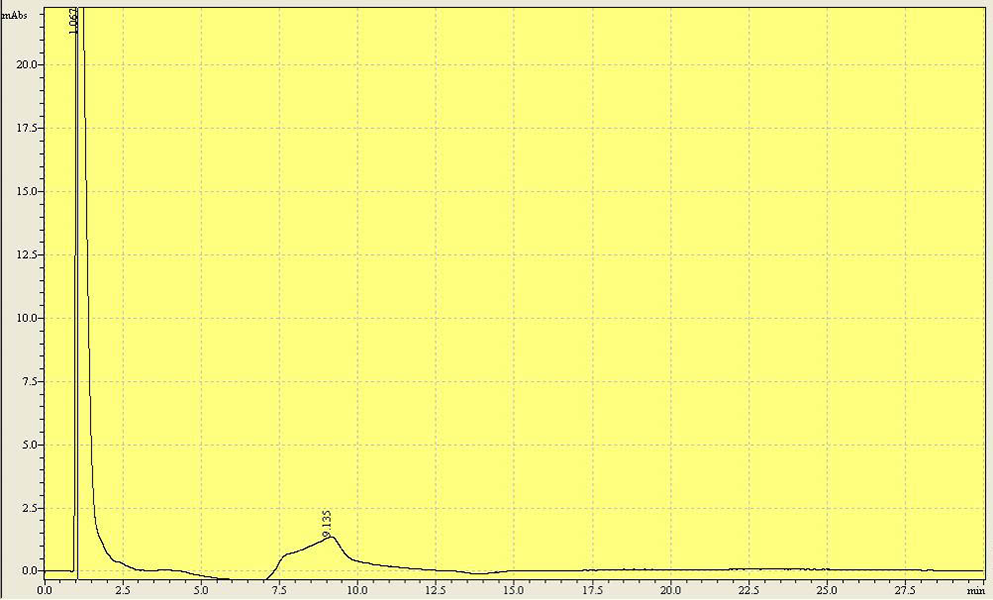

Supplement: S4 Fig — Liquid chromatogram of a 1 ppm desmethyl-bromethalin standard showing detection at 9.135 minutes. (TIF) [file pone.0213248.s004.tif]

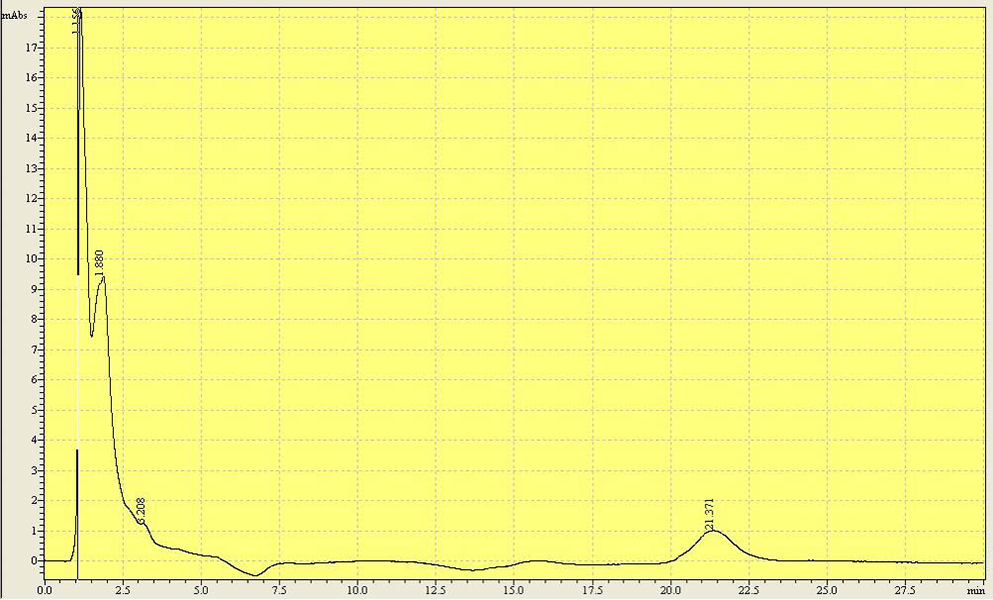

Supplement: S5 Fig — Liquid chromatogram of a 1 ppm bromethalin standard showing detection at 21.371 minutes. (TIF) [file pone.0213248.s005.tif]

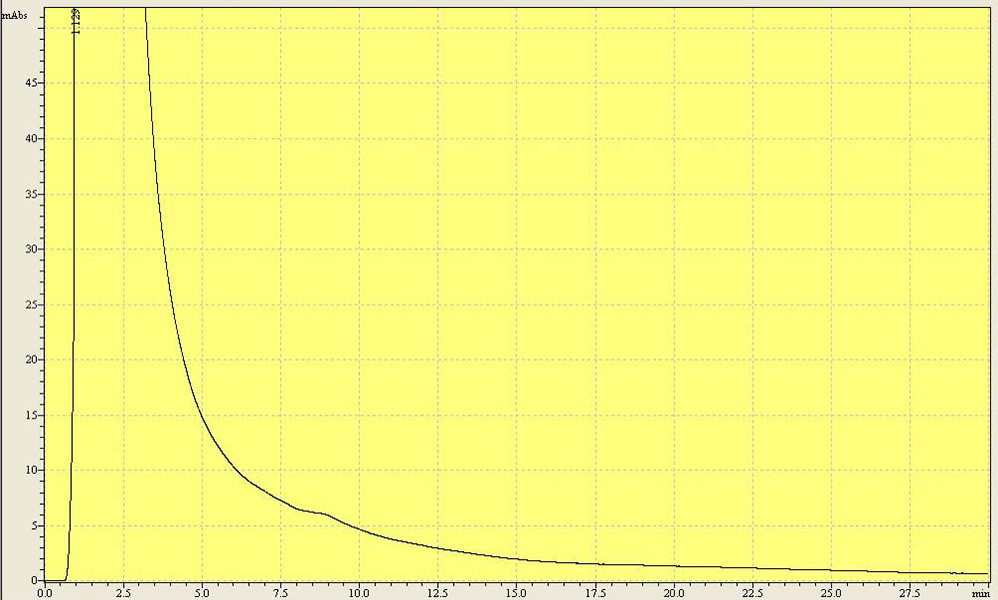

Supplement: S6 Fig — Liquid chromatogram of a control fecal sample showing no detection at either the desmethyl-bromethalin or bromethalin retention times. (TIF) [file pone.0213248.s006.tif]

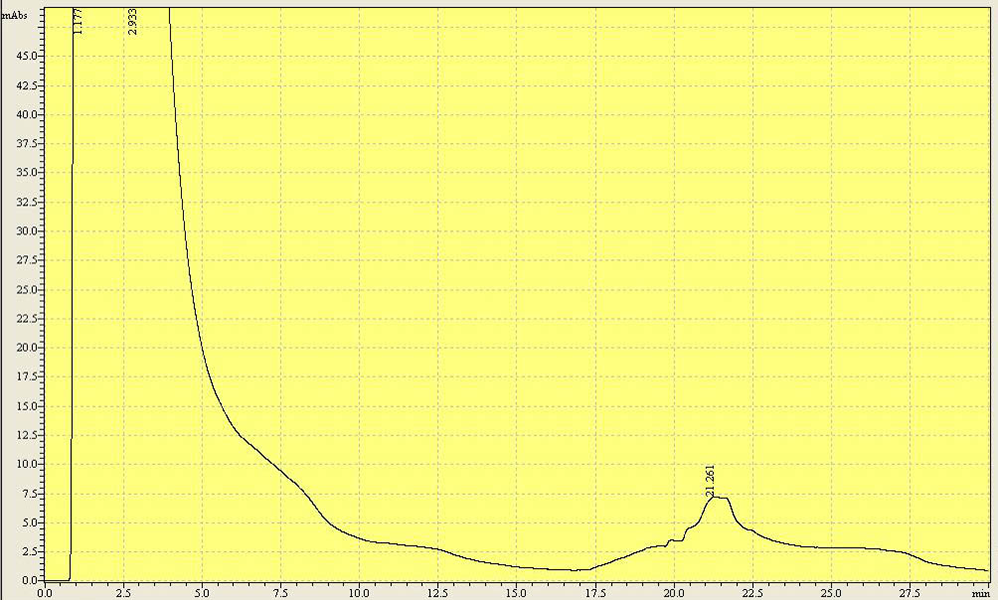

Supplement: S7 Fig — Liquid chromatogram of fecal sample from subject 1 showing detection of bromethalin at 21.261 minutes. (TIF) [file pone.0213248.s007.tif]

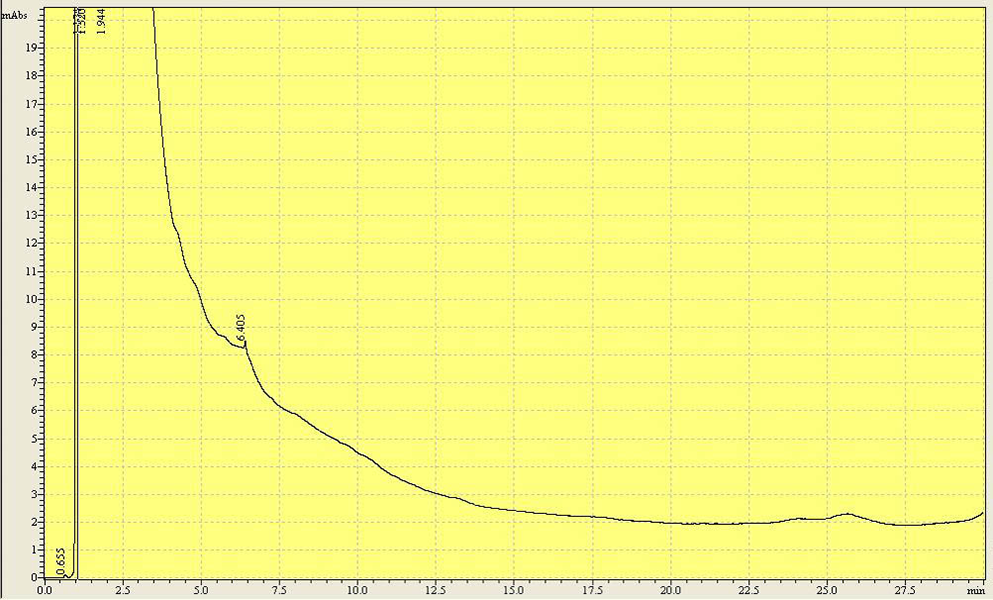

Supplement: S8 Fig — Liquid chromatogram of a chicken brain sample showing no detection at either the desmethyl-bromethalin or bromethalin retention times. (TIF) [file pone.0213248.s008.tif]

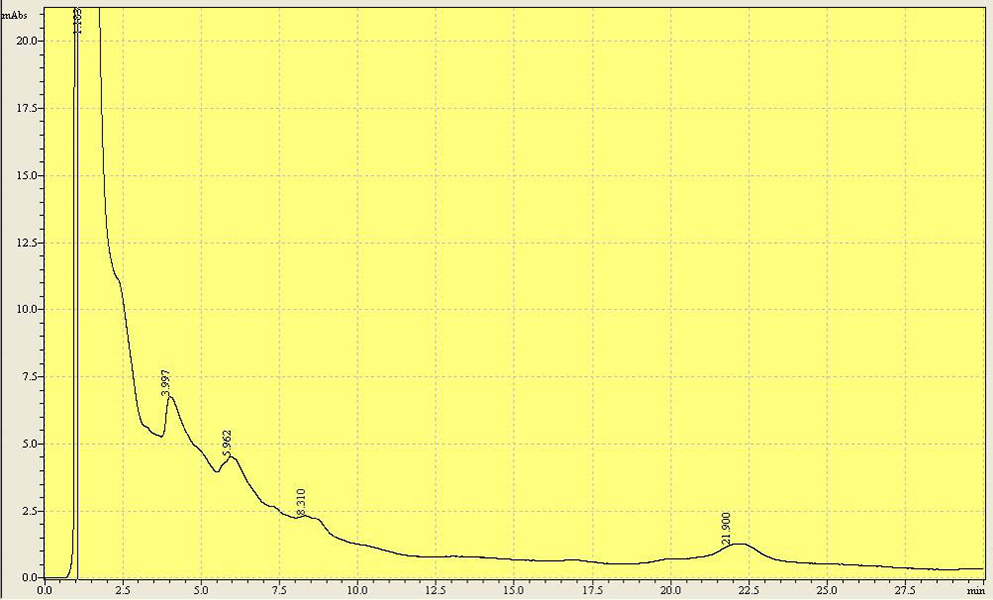

Supplement: S9 Fig — Liquid chromatogram of a brain sample from subject 4 showing detection of bromethalin at 21.9 minutes. (TIF) [file pone.0213248.s009.tif]

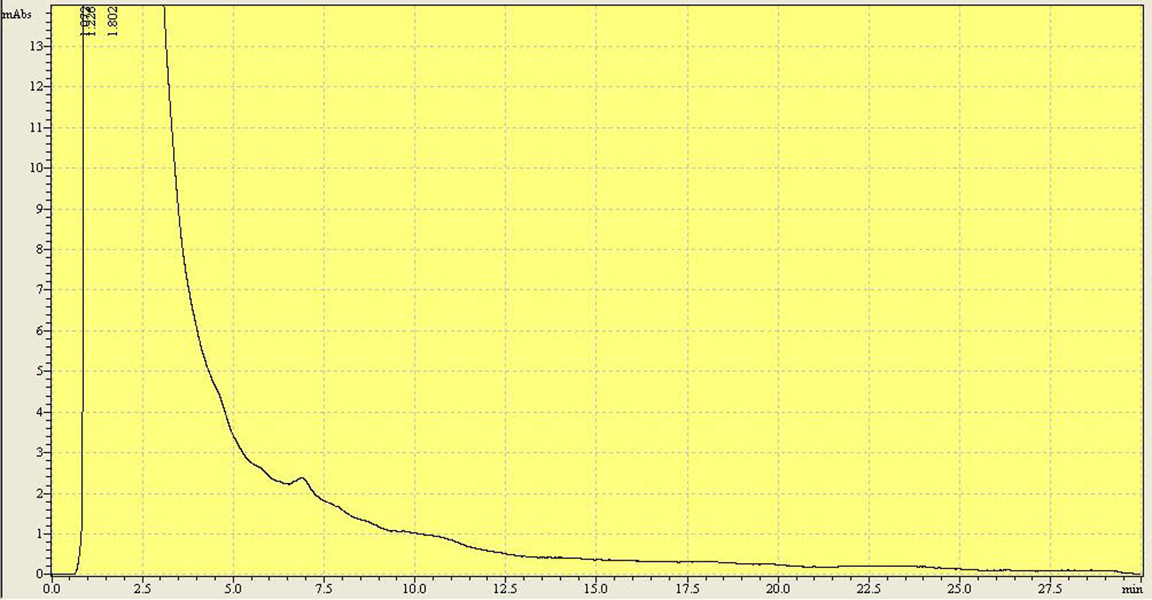

Supplement: S10 Fig — Liquid chromatogram of a chicken liver sample showing no detection at either the desmethyl-bromethalin or bromethalin retention times. (TIF) [file pone.0213248.s010.tif]

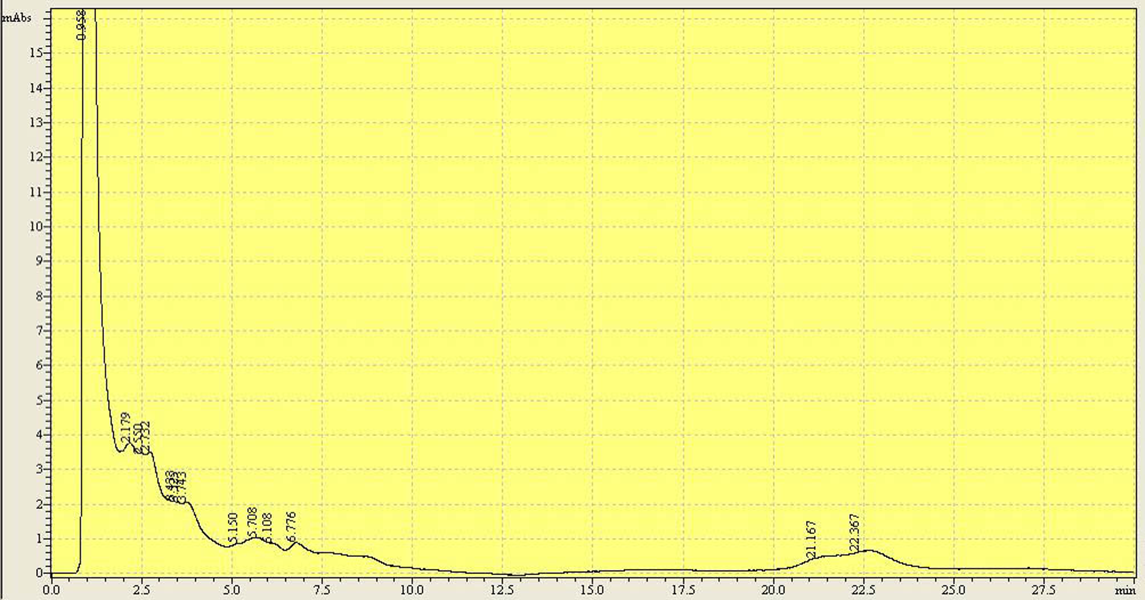

Supplement: S11 Fig — Liquid chromatogram of a liver sample from subject 4 showing detection of bromethalin at 21.17 minutes. (TIF) [file pone.0213248.s011.tif]
